# Essentials of Orthodontia

**Published:** 1914-10-15

**Authors:** 


ESSENTIALS OF ORTHODONTIA. This is a little book of 100 pages by Dr. Van Broadus Dalton, Professor of Orthodontia in the Ohio College of Dental Surgery. It does not pretend to be a treatise or text work on this subject nor is it intended to supplant the existing more comprehensive books on this subject. It is really a text manual on the more technical features of the subject and is designed to assist college students in acquiring a systematic technique that is practicable within the time devoted to this subject by most dental schools. It could be used to advantage by the practitioner who wants to know the technical methods involved in orthodontic practice without a more serious study of the science and art involved in a comprehensive knowledge of the subject. It is published by P. Blakiston Son & Co., Philadelphia.

